# Using Social Media as a Survey Recruitment Strategy for Post-Secondary Students During the COVID-19 Pandemic

**DOI:** 10.1177/00469580211059305

**Published:** 2021-12-13

**Authors:** Simran Purewal, Paola Ardiles, Erica Di Ruggiero, John Vincent L. Flores, Sana Mahmood, Hussein Elhagehassan

**Affiliations:** 1 241829 Faculty of Health Sciences, Simon Fraser University, Burnaby, BC, Canada; 2 274071 Dalla Lana School of Public Health, University of Toronto, Toronto, ONT, Canada; 3 98586 Faculty of Arts and Science, University of Toronto, Toronto, ONT, Canada

**Keywords:** COVID-19, coronavirus, social media, survey recruitment, post-secondary students

## Abstract

The COVID-19 pandemic rapidly forced Canadian post-secondary students into remote learning methods, with potential implications on their academic success and health. In recent years, the use of social media to promote research participation and as a strategy for communicating health messages has become increasingly popular. To better understand how the pandemic has impacted this population, we used social media platforms to recruit students to participate in a national bilingual COVID-19 Health Literacy Survey. The purpose of the survey was to assess the health literacy levels and online information-seeking behaviors of post-secondary students in relation to the coronavirus. This paper outlines the social media recruitment strategies used for promoting participation in the survey among Canadian post-secondary students during the pandemic. Facebook, Twitter, and Instagram accounts were created to promote the online survey. The objective of this paper is to examine the use of Instagram, Facebook, and Twitter as survey recruitment strategies tailored to students. Data analytics from these platforms were analyzed using descriptive statistics. We found that the most commonly used platform for survey dissemination was Twitter, with 64800 total impressions recorded over 3 months. The use of social media as a survey recruitment strategy showed promise in the current context of COVID-19 where many students are participating in online learning and for a study population that actively uses these platforms to seek out information.

## Highlights

**1. What do we already know about this topic?**

The use of social media to promote research participation is feasible given the widespread use of technology and has become increasingly popular within recent years, especially among younger demographics.

**2. How does your research contribute to the field?**

This paper examines the use of social media platforms to enhance research participation among post-secondary students in the current context of COVID-19, in which traditional recruitment methods can be difficult to implement.

**3. What are your research’s implications toward theory, practice, or policy?**

This research will be of particular interest to public health practitioners and scholars, particularly those in the field of health communication as this paper highlights the use of social media platforms to create tailored health messages to post-secondary students.

## Introduction

On January 25, 2020, the first case of the novel coronavirus (COVID-19) was detected in Canada.^1^ By March 28, 2020, the Federal Government announced a ban on air travel for individuals with COVID-19 symptoms, while provinces and territories established stay-at-home orders.^2^ During this time, post-secondary schools rapidly suspended in-person classes, forcing students into remote learning methods to curb the spread of COVID-19. The prompt implementation of public health measures, including physical distancing and stay-at-home orders, made it more difficult to carry out health research in part due to the challenges associated with recruiting participants online.

Health literacy is defined as the “degree to which individuals can access, understand, evaluate, and communicate information to engage with the demands of different health contexts in order to promote and maintain good health across the life-course”.^3^ It is crucial when navigating the Internet to identify, comprehend, and apply COVID-19-related information in one’s daily life. In May 2020, the COVID-Health Literacy Consortium was established to examine the health literacy levels of students during the pandemic and draw attention to how digital literacy is significant for searching for online health information.^4^ To better understand Canadian post-secondary students' health literacy levels, their online information-seeking behaviors, and how the pandemic has impacted their health and well-being, a bilingual COVID-19 Health Literacy Survey was initiated during the first wave of COVID-19 in Canada.

Amid the ongoing pandemic, there has been a drastic increase in the use of technology, especially social media, to connect with others and engage in virtual interactions.^3^ Social media has also become a significant platform for sharing information pertaining to COVID-19 by governments, public health agencies, and post-secondary institutions.^4^ For instance, the World Health Organization and the Public Health Agency of Canada regularly disseminate coronavirus-related content through their Twitter accounts.^5^ These organizations play an important role in sharing credible, timely public health communications. Furthermore, students frequently turn to social media platforms, like Facebook, to seek out information on a daily basis.^6^ In light of the logistical challenges presented by COVID-19, including the shutdown of on-campus events, we opted to disseminate the COVID-19 Health Literacy Survey online. We primarily used 3 social media platforms for survey dissemination: Twitter, Instagram, and Facebook. These channels were selected in order to reach a wide range of post-secondary students based on the research team’s experience using social media. Most students are also active on multiple social media sites, potentially making a multi-channel approach to survey recruitment more effective.^7,8^

The use of social media as a survey recruitment method gained popularity in recent years and has become more prominent during the COVID-19 pandemic, in which traditional recruitment methods, such as in-person recruitment, are less feasible.^8^ Using social media to promote survey participation is a low-cost strategy that allows researchers to reach a wide range of people across geographic boundaries.^9^ Ad campaigns to promote participation vary by study, but campaigns typically involve images, keywords related to the study, written descriptions about the project’s scope, and are tailored to the population of interest.^10,11^ In past studies, many researchers have opted to disseminate surveys using their personal social media accounts, while others have created new accounts specific to the research project.^10^ Furthermore, it is increasingly common for researchers to use paid advertising on Facebook and Instagram to recruit young adults to participate in surveys.^12,13^ Several studies also highlight incentives in their campaigns, like gift vouchers, to promote participation.^12^ These campaigns are regularly monitored throughout the study’s duration, and the research team may adjust their recruitment approach based on the number of respondents.

The objective of this paper is to examine the use of Instagram, Facebook, and Twitter as survey recruitment strategies tailored to students to encourage them to take part in an online COVID-19 Health Literacy Survey in Canada.

## Methods

### Recruitment

**Figure 1.**
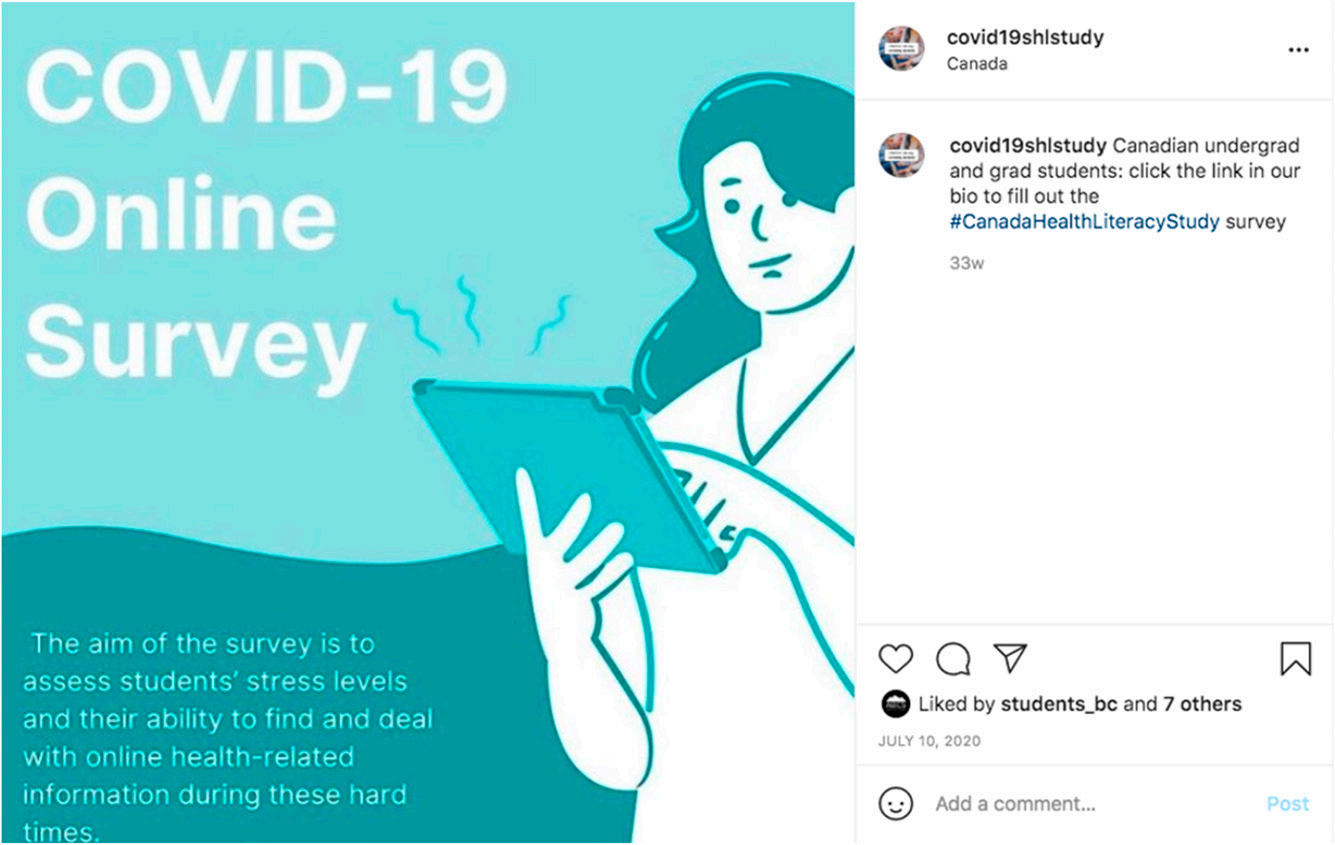
Instagram post from @COVID19SHLStudy.

**Figure 2.**
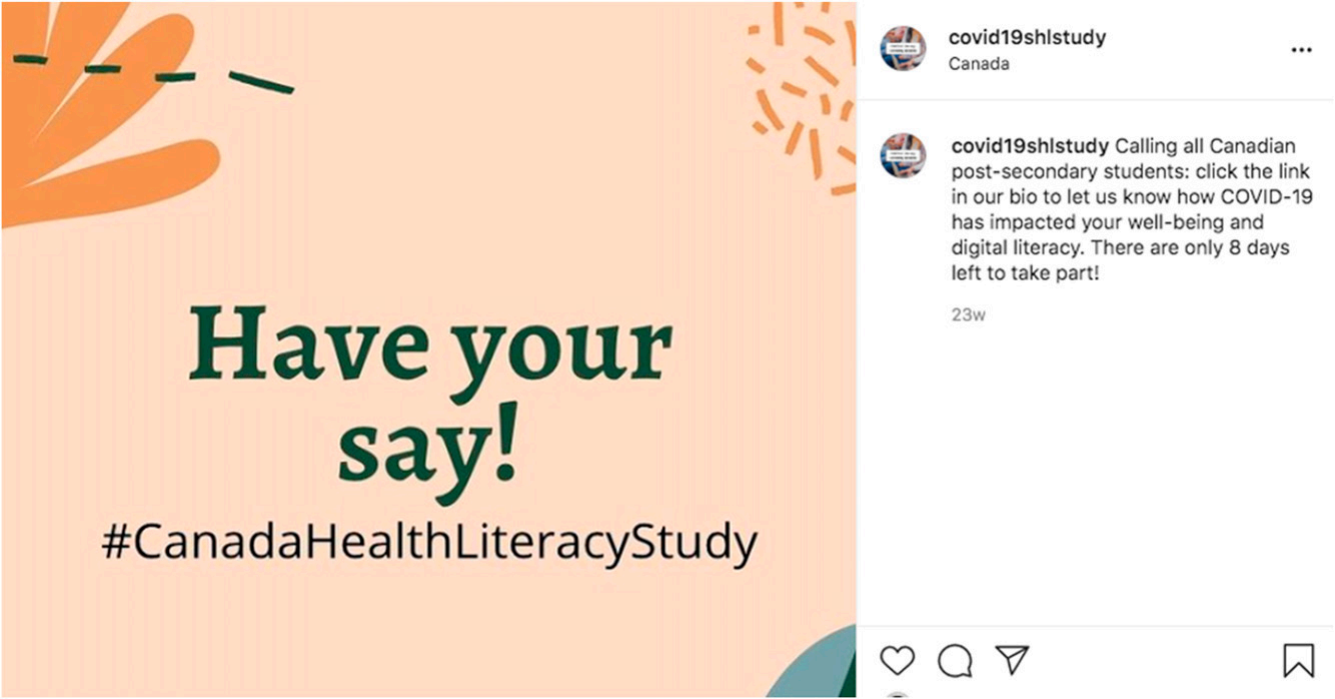
Instagram post from @COVID19SHLStudy (English).

**Figure 3.**
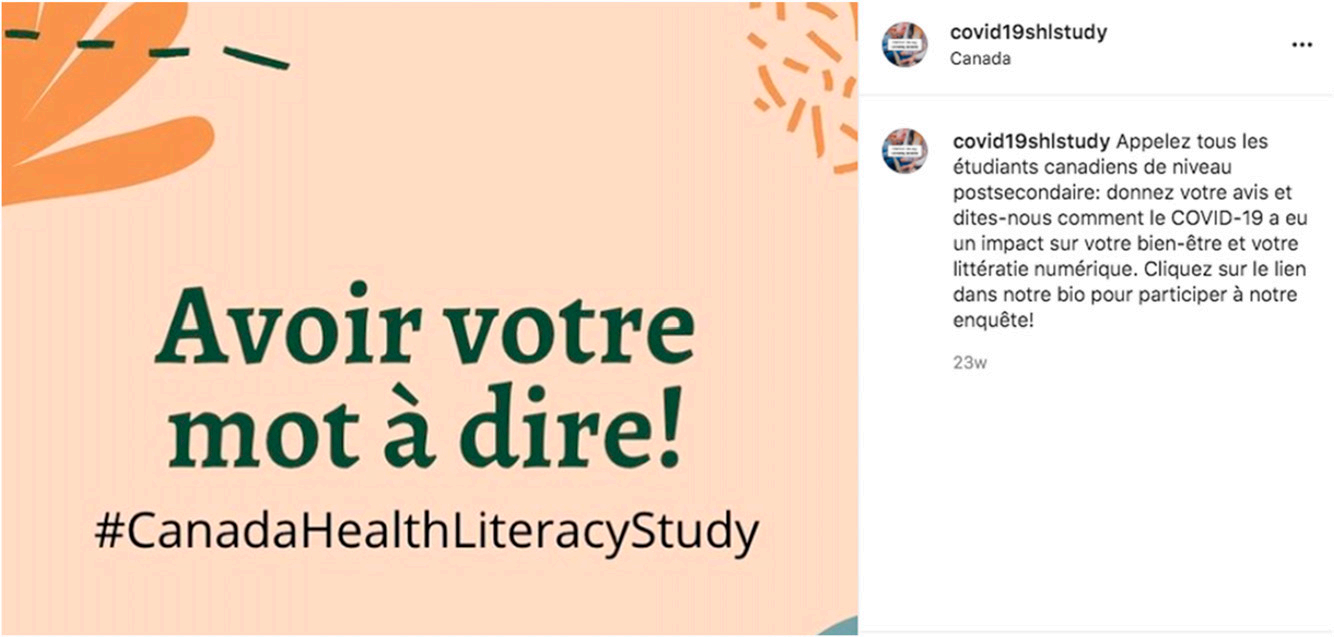
Instagram post from @COVID19SHLStudy (French).

This paper outlines the methodology of creating social media accounts and recruiting students. The English version of COVID-19 Health Literacy Survey, open to all post-secondary students in Canada, launched on July 1, 2020. The French version of the survey launched at a later date on July 14, 2020. The study received ethics approval from the University of Toronto’s Health Sciences Research Ethics Board (reference number 39254), and subsequently Simon Fraser University’s Health Research Ethics Board (reference number 20200186). Consent to participate in the survey was obtained from an informed consent found on the first page of the survey. Participants were also provided a list of resources about mental health and COVID-19. We used the Qualtrics XM platform to administer and disseminate the online survey. In addition to email requests sent to student unions and communication departments at post-secondary institutions, Instagram, Facebook, and Twitter accounts specific to the study were created to recruit students. Tables 1–3 illustrate the specific recruitment methods used on each platform. To create informative social media posts and engage with participants on these platforms, we used *Canva*, a graphic design platform. These posts were tailored to students and available in both English and French since we promoted a bilingual survey (Figures 1‐3). In order to share the English and French survey links, a Linktree containing both versions of the survey was distributed on each platform. We also generated 2 hashtags (#CanadaHealthLiteracySurvey and #CanadaHealthLiteracyStudy) in order to group posts related to the survey. Tweets involving these hashtags provided social media users with information about the study and a direct link to participate in the survey. To track the level of content engagement, social media data analytics on each platform were analyzed on a weekly basis.

**Table 1.** Twitter Recruitment Flowchart.

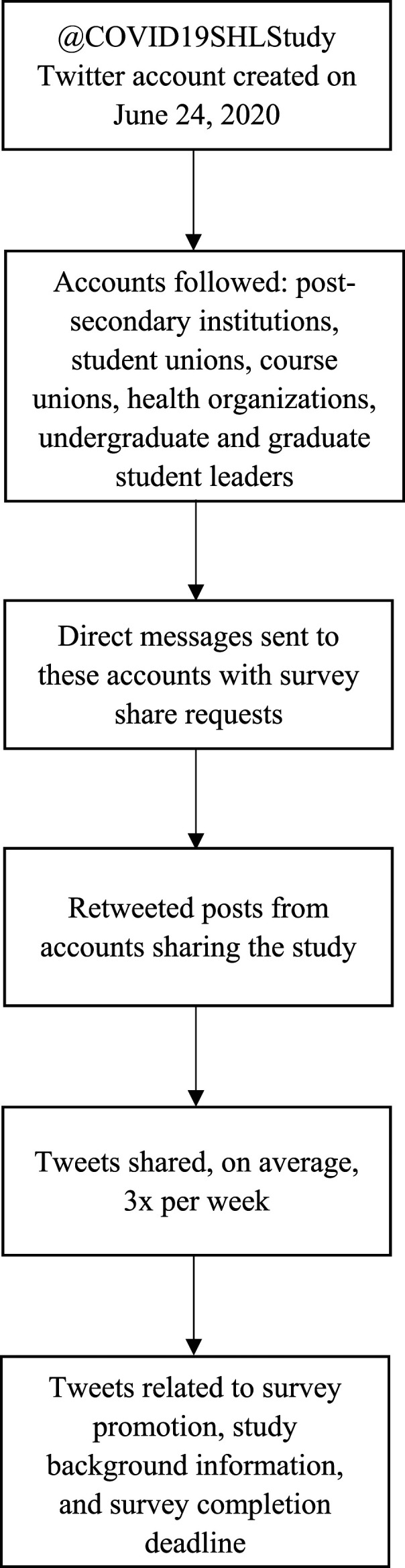

**Table 2.** Instagram Recruitment Flowchart.

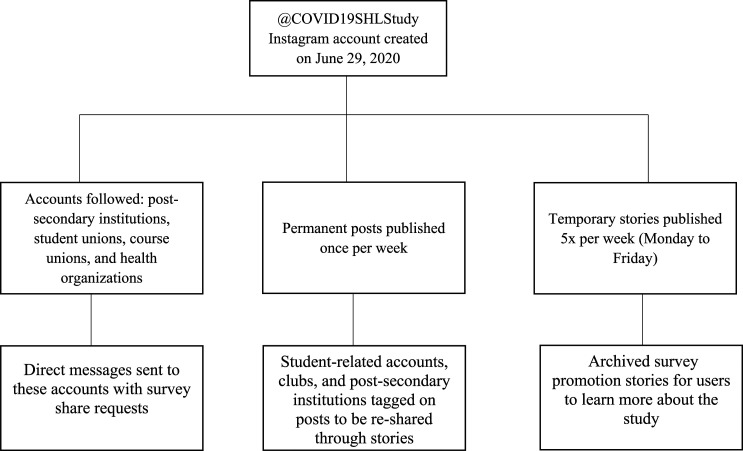

**Table 3.** Facebook Recruitment Flowchart.

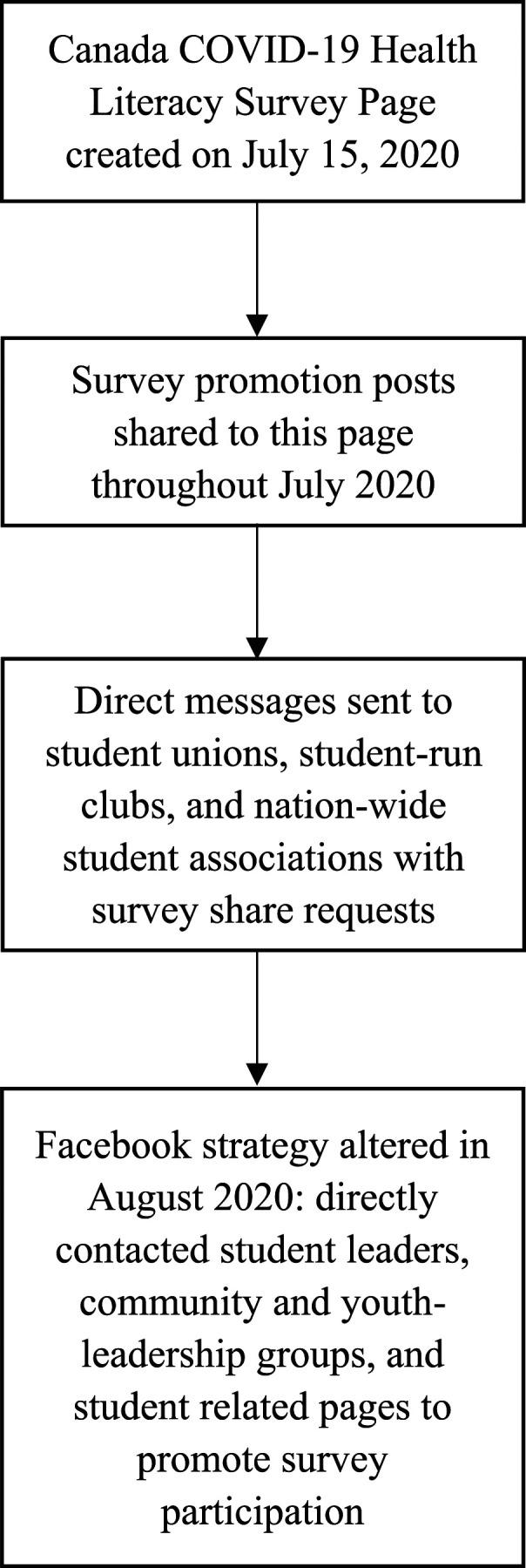

The first wave of followers on each social media platform was recruited using a multi-pronged approach. At the outset, we identified accounts related to higher education in Canada, such as the official social media accounts of post-secondary institutions (eg, @SimonFraserU) and accounts representing nation-wide student unions (eg, @Casaacae). We then examined their following lists to discover student-run clubs and associations within each institution, followed these accounts, and directly messaged them to provide background information about the study. The accounts interested in taking part in the study subsequently followed our accounts.

## Results

In total, 2679 participated in our online survey. Across 3 social media platforms, we garnered a total of 69 866 impressions. By the survey closing date, there were a total of 259 posts containing the survey’s hashtags (#CanadaHealthLiteracyStudy and #CanadaHealthLiteracySurvey) on all of the platforms. Furthermore, 94 universities and colleges across Canada agreed to share the survey on their respective social media platforms. Of the institutions that promoted the survey, 63 were universities and 31 were colleges. Ontario had the highest number of participating institutions as 22 universities and 9 colleges opted to share the survey, followed by British Columbia, with 19 participating post-secondary schools. These 2 provinces are locations for the 2 universities that led the study. On the 3 social media sites, a total of 54 course unions and 31 undergraduate and graduate student unions promoted the study, making course unions at post-secondary institutions the most active group with regard to survey promotion.

### Twitter

By September 30th, the account had 66 followers and 316 tweets. A total of 192 tweets contained #CanadaHealthLiteracySurvey or #CanadaHealthLiteracyStudy. Additionally, the total number of survey link clicks from our tweets was 160. Based on our analysis, Twitter was the most popular platform for survey promotion as this platform resulted in 64 800 impressions, which refers to the number of times Twitter users saw our content. Moreover, we reached a total of 1199 direct profile visits throughout the study duration. The primary accounts that promoted the survey were course unions at universities (11), student clubs and groups that address topics such as physical health, mental health, and wellness, (10), and undergraduate student unions at universities (9).

### Instagram

The survey’s Instagram account reached 109 followers and a total of in 30 posts. Furthermore, there were an additional 37 posts containing #CanadaHealthLiteracyStudy and #CanadaHealthLiteracySurvey. On this platform, 1909 accounts were contacted through direct messaging to promote the survey. Of this, 351 Instagram pages posted a temporary story related to the survey, and 44 accounts shared a permanent post on their page. Over the duration of 3 months, our account gained 375 profile visits and reached 3484 users across the platform. Instagram was the second most popular platform for survey dissemination with a total of 4151 impressions. There were also 208 survey link clicks from our page, and our posts were re-shared by other accounts a total of 155 times. Most commonly, course unions at universities promoted the survey on Instagram (16), followed by student clubs and groups (7), and health and wellness pages at post-secondary institutions.

### Facebook

Facebook was a frequently used platform for survey promotion among undergraduate student groups. A total of 216 Facebook pages and users were contacted via direct message to promote the survey. Of this, 72 organizations shared posts pertaining to the survey on their Facebook page and an additional 4 pages created temporary stories to promote the survey. In total, there were 30 posts containing the hashtags #CanadaHealthLiteracySurvey or #CanadaHealthLiteracyStudy. Course unions at universities (24), undergraduate student unions (12), and mental health-related organizations (10) most frequently promoted the survey on Facebook. Based on the data analytics provided by Facebook Insights, our posts reached 915 users and 67 engagements (post clicks, reactions, comments, and shares).

## Discussion

Based on the results of this study, the use of social media to promote survey participation is feasible in the current context of COVID-19, in which many individuals are studying remotely, making traditional recruitment methods less practical. Our research team included several post-secondary students, which informed our decision to use social media as a recruitment strategy. Furthermore, each member of the team had extensive experience using these online platforms, allowing us to create tailored posts that aligned with the demographic groups we were attempting to reach.

Over the duration of the study, our social media posts garnered a total of 69 866 impressions across 3 platforms. Our team also elected to create new social media accounts pertaining to the study. Although study-specific accounts may take longer to gain a significant following, the creation of study-specific Twitter and Instagram accounts allowed us to create content centered on survey promotion, network with health and student-oriented accounts to reach participants, and track levels of engagement. Despite the widespread use of Facebook for health communication and recruitment, we found that Twitter was the most popular platform for dissemination of our survey.^13^ In addition, we found the use of newly developed hashtags relevant to the survey to be an engaging way to group and refer to promotional posts across all platforms. The use of social media to recruit survey participants is relatively new in health research; however, several studies have demonstrated the effectiveness of this method, particularly at reaching youth participants.^12,14,15^ Furthermore, utilizing a multi-pronged strategy that relied on various social media platforms potentially enabled us to connect with a wider audience.

Throughout the duration of the study, we found that course unions at universities were most likely to distribute the survey. This could be due to the potential to better connect with students on a smaller scale, such as a course-specific setting, as opposed to a much broader institution-wide student union. We also observed that Ontario and British Columbia had the highest number of post-secondary institutions that agreed to share and disseminate the survey. This could be attributed to the fact that the principal investigators and the research team were based at post-secondary institutions in these 2 provinces and were more recognizable to potential participants in these locations.

Although 2679 students completed our survey, our analysis involving both complete and incomplete survey responses demonstrates that we reached a total of 3252 students. We evaluated the potential effectiveness of our recruitment strategies based on a review of the literature highlighting characteristics of strong recruitment strategies using social media. Some of the characteristics included evidence of a large, diverse sample of participants and the ability to gain responses from hard-to-reach populations.^14-16^ While studies examining the efficacy of recruiting respondents using social media are limited, we were able to effectively recruit a large sample of Canadian post-secondary students across 10 provinces during the COVID-19 pandemic over a short period of time.

While our study did not seek to explore how health messages can be communicated via social media during a pandemic, it does demonstrate how a combination of social media platforms hold potential to reach post-secondary students. Recent studies also show that post-secondary students commonly access COVID-19 information from social media.^11,15^ Communicating reliable information online is especially important as the overwhelming amount of information readily available online concerning COVID-19 has resulted in an information epidemic, more commonly referred to as an infodemic.^17^ Infodemics can threaten compliance with public health actions, like stay-at-home orders.^18^ Based on this, post-secondary institutions and public health agencies play an important role in mitigating misinformation, particularly among students who may be concerned about media exposure to the pandemic and worried about their eventual return to in-person classes.^19^ Additionally, communicating health messages through social media platforms increases accessibility to vital COVID-19 information that is required for understanding and increasing the uptake of health protective behaviors, like mask-wearing.^19^

### Strengths

This paper contributes to the growing literature regarding the use of social media to recruit survey participants. Using social media as a survey recruitment strategy is feasible especially during the ongoing COVID-19 pandemic. It is a low-cost strategy that allows researchers to create tailored content and posts according to the study population.^9^ The use of internet-based recruitment methods also enables researchers to reach participants across a country like Canada with a vast geography and in the face of potential time constraints.^9^ Moreover, the authors used a unique approach to promote study participation as social media accounts specific to the study were created on 3 platforms. We chose to employ a social media strategy to recruit participants, which had not been used by other researchers in the International Health Literacy Consortium. Furthermore, the research team was mainly composed of undergraduate students at different academic institutions, who use social media for leisure and academic purposes, providing them with a sound understanding of how to engage with students on various platforms. As such, we were able to recruit more than 2600 students to take part in an online survey during the first wave of COVID-19 in Canada. This paper also analyzes the combined use of 3 social media platforms to promote survey participation among students, which has not yet been studied extensively.^20,21^ Additionally, the authors directly contacted student unions and leaders within the institution, as opposed to relying on more passive broad mailing lists or email requests. While many past studies have used one social media platform, namely, Facebook, to reach students, our study relied on a multi-pronged approach to recruit students on various social media sites.^8,13,21^ We also used Twitter as a key recruitment platform due to the network of academics and students active on this site, which is not common in previous studies.

Social media platforms, like Facebook, Instagram, and Twitter, provide access to user insights in order to determine the levels of content engagement with posts. Researchers and public health agencies can use these analytics to identify patterns of engagement and areas of improvement when sharing information online.

### Limitations

While this survey recruitment strategy was feasible given the context of the COVID-19 pandemic, we were unable to determine the distribution of participants across our 3 social media platforms. Because we did not utilize paid advertising on these platforms, we may have limited the reach of our posts. Furthermore, we did not measure how many views were required to successfully recruit one participant or the number of target users reached on social media, but these are important considerations to incorporate in future studies. Moreover, social media as a recruitment tool is still fairly novel, which may raise concerns regarding the lack of regulatory guidance of this strategy.^17^ There were also a significant number of surveys ongoing at this time, potentially resulting in survey fatigue.^3^ Similarly, we noticed that a majority of student-run health organizations responded to the survey requests given the health literacy focus; therefore, we may be missing responses from students outside of this discipline. Although the findings are not generalizable, this study provides insight into the use of multiple social media as a survey recruitment strategy, which is particularly useful for conducting research during the COVID-19 pandemic.

### Implications

Based on our findings, researchers may find it beneficial to create study-specific accounts on various social media platforms to engage with their study population through an accessible online medium. Furthermore, the mass amount of online information pertaining to COVID-19 requires governments and public health agencies to play an active role in mitigating misinformation. One way to curb the spread of misinformation is through social media platforms to engage with younger audiences, address misconceptions, and better comprehend how this population accesses and understands coronavirus-related information.^22,23^ Moreover, while Facebook and Instagram are widely used to recruit participants, we found that some student unions and clubs opted to share the survey on Discord, an online messaging and distribution platform that has become increasingly popular during the pandemic.^24^ Future studies should explore the use of newer social media platforms, like Discord, to distribute and promote surveys in an online environment.

## Conclusion

This study examined the use of social media as a survey recruitment tool among post-secondary students. Based on our findings, promoting surveys on social media platforms is a feasible recruitment strategy for research conducted in the context of COVID-19 and relevant to the demographic groups we attempted to reach. Utilizing a multi-channel approach also allowed us to reach a greater number of students active on various platforms. Building on our insights, public health agencies and post-secondary institutions should further explore these platforms to create and publish timely and accurate COVID-19 messages to further curb the spread of misinformation about COVID-19 among young adults in higher education.

## References

[1] Canadian Healthcare Network . COVID-19: A Canadian Timeline. Canadianhealthcarenetwork.ca. https://www.canadianhealthcarenetwork.ca/covid-19-a-canadian-timeline. Accessed January 12, 2021.

[2] The Canadian Press . A Timeline of Events in Canada’s Fight against COVID-19. Thestar.com. Published June 18, 2020 https://www.thestar.com/news/canada/2020/06/18/a-timeline-of-events-in-canadas-fight-against-covid-19.html. Accessed January 12, 2021.

[3] Mitic W Rootman I . An Inter-sectoral Approach for Improving Health Literacy for Canadians. Victoria, BC: Public Health Association of British Columbia; 2012:4. https://nccdh.ca/resources/entry/an-inter-sectoral-approach-for-improving-health-literacy. Accessed October 20, 2020.

[4] Dadaczynski K Okan O Messer M , et al. Digital health literacy and web-based information-seeking behaviors of university students in Germany during the COVID-19 Pandemic: cross-sectional survey study. JMIR Public Health Surveill. 2021;23(1):e24097. https://www.jmir.org/2021/1/e24097/. Accessed February 1, 2021. 10.2196/24097 PMC7813561 33395396

[5] Drouin M McDaniel BT Pater J Toscos T . How parents and their children used social media and technology at the beginning of the COVID-19 pandemic and associations with anxiety. Cyberpsychol Behav Soc Netw. 2020;23(11):727-736. https://www.liebertpub.com/doi/10.1089/CYBER.2020.0284. Accessed February 1, 2021. 32726144 10.1089/cyber.2020.0284

[6] Tsao SF Chen H Tisseverasinghe T Yang Y Li L Butt ZA . What social media told us in the time of COVID-19: a scoping review. Lancet Digit Health. 2021;3(3):e175-e194. https://www.thelancet.com/journals/landig/article/PIIS2589-7500(20)30315-0/fulltext. Accessed February 1, 2021. 33518503 10.1016/S2589-7500(20)30315-0 PMC7906737

[7] Wang Y Hao H Platt LS . Examining risk and crisis communications of government agencies and stakeholders during early-stages of COVID-19 on Twitter. Comput Human Behav. 2021;114:106568. https://pubmed.ncbi.nlm.nih.gov/32982038/. Accessed February 3, 2021. 32982038 10.1016/j.chb.2020.106568 PMC7508681

[8] Kühne S Zindel Z . Using Facebook and Instagram to recruit web survey participants: a step by-step guide and application. Survey Methods. 2020. https://surveyinsights.org/?p=13558. Accessed September 5, 2021.

[9] Harfield S Elliott S Ramsey L Housen T Ward J . Using social networking sites to recruit participants: methods of an online survey of sexual health, knowledge and behaviour of young South Australians. Aust N Z J Publ Health. 2021;45(4):348-354. [Internet] https://onlinelibrary.wiley.com/doi/abs/10.1111/1753-6405.13117. Accessed September 5, 2021. 10.1111/1753-6405.13117 34097339

[10] Gelinas L Pierce R Winkler S Cohen IG Lynch HF Bierer BE . Using social media as a research recruitment tool: ethical issues and recommendations. Am J Bioeth. 2017;17(3):3-14. doi:10.1080/15265161.2016.1276644. https://www.ncbi.nlm.nih.gov/pmc/articles/PMC5324729/. Accessed December 19, 2020. PMC5324729 28207365

[11] Frederick A Run Y . Social media usability among university students: a case study of Jiangsu University. Global Media J. 2018;16(31):1-8. http://www.globalmediajournal.com/open-access/social-media-usability-among-university-student-a-case-study-of-jiangsu-university-china.php?aid=87199. Accessed February 1, 2021.

[12] Nagel T Remillard C Aucoin R Takenishi A . Findings on student use of social media at the collegiate, undergraduate, and graduate levels: implications for post-secondary educators. J Univ Teach Learn Pract. 2018;15(1). https://files.eric.ed.gov/fulltext/EJ1173809.pdf. Accessed February 6, 2021.

[13] Kayrouz R Dear BF Karin E Titov N . Facebook as an effective recruitment strategy for mental health research of hard to reach populations. Internet Interv. 2016;4:1-10. https://www.ncbi.nlm.nih.gov/pmc/articles/PMC6096235/. Accessed December 20, 2020. 30135786 10.1016/j.invent.2016.01.001 PMC6096235

[14] Ali SH Foreman J Capasso A Jones AM Tozan Y DiClemente RJ . Social media as a recruitment platform for a nationwide online survey of COVID-19 knowledge, beliefs, and practices in the United States: methodology and feasibility analysis. BMC Med Res Methodol. 2020;20(1):116. https://bmcmedresmethodol.biomedcentral.com/articles/10.1186/s12874-020-01011-0. Accessed December 19, 2020. 32404050 10.1186/s12874-020-01011-0 PMC7220591

[15] Ford KL Albritton T Dunn TA Crawford K Neuwirth J Bull S . Youth study recruitment using paid advertising on Instagram, Snapchat, and Facebook: Cross-sectional survey study. JMIR Public Health and Surveill. 2019;5(4):e14080. 10.2196/14080 PMC6811770 31599739

[16] McRobert CJ Hill JC Smale T Hay EM Van Der Windt DA . A multi-modal recruitment strategy using social media and internet-mediated methods to recruit a multidisciplinary, international sample of clinicians to an online research study. PLoS One. 2018;13(7):e0200184. https://journals.plos.org/plosone/article?id=10.1371/journal.pone.0200184. Accessed December 19, 2020. 29979769 10.1371/journal.pone.0200184 PMC6034855

[17] World Health Organization . Managing the COVID-19 Infodemic: Promoting Healthy Behaviours and Mitigating the Harm from Misinformation and Disinformation. Who.com; 2020. Published on September 23 https://www.who.int/news/item/23-09-2020-managing-the-covid-19-infodemic-promotin. Accessed January 10, 2021.

[18] Galvão J . COVID-19: the deadly threat of misinformation. Lancet Infect Dis. 2020;21(5):e114. https://www.thelancet.com/journals/lanres/article/PIIS1473-3099(20)30721-0/fulltext. Accessed January 10, 2021. 33031753 10.1016/S1473-3099(20)30721-0 PMC7535539

[19] Schiff M Zasiekina L Pat-Horenczyk R Benbenishty R . COVID-related functional difficulties and concerns among university students during COVID-19 pandemic: a binational perspective. J Community Health 2020;1-9. Accessed January 7, 2021. 33029678 10.1007/s10900-020-00930-9 PMC7540431

[20] King D O’Rourke N DeLongis A . Social media recruitment and online data collection: a beginner’s guide and best practices for accessing low-prevalence and hard-to-reach populations. Can Psychol. 2014;55:240-249. http://psycnet.apa.org/journals/cap/55/4/240/. Accessed January 22, 2021.

[21] Whitaker C Stevelink S Fear N . The use of Facebook in recruiting participants for health research purposes: a systematic review. J Med Internet Res. 2017;19(8):e290. https://www.jmir.org/2017/8/e290/. Accessed September 7, 2021. 28851679 10.2196/jmir.7071 PMC5594255

[22] Olaimat AN Aolymat I Shahbaz HM Holley RA . Knowledge and information sources About COVID-19 among university students in Jordan: a cross-sectional study. Frontiers in Public Health. 2020;8:254. https://pubmed.ncbi.nlm.nih.gov/32574314/, https://www.ncbi.nlm.nih.gov/pmc/articles/PMC7540431/. Accessed on January 8, 2021, Accessed January 15, 2021. 32574314 10.3389/fpubh.2020.00254 PMC7274134

[23] Hauer MK Sood S . Using social media to communicate sustainable preventive measures and curtail misinformation. Front Psychol. 2020;11:568324. https://www.frontiersin.org/articles/10.3389/fpsyg.2020.568324/full. Accessed January 28, 2021. 33178073 10.3389/fpsyg.2020.568324 PMC7597381

[24] Discord . Your Place to Talk and Hang Out. Discord.com. https://discord.com/. Accessed on February 26, 2021.

